# Effects of Rope Therapy on Social Attention and Temperament Traits in Autistic Children

**DOI:** 10.3390/children12070881

**Published:** 2025-07-03

**Authors:** Mi Zhou, Kevin Hung, Marco Chun-Cheong Wong, Tony Keng-Tou Chau, Benny Wai-Lun Lam, Cecilia Yuen-Ching Chu, Jialiang Gu, Jiawen Dai, Daniel Hung-Kay Chow

**Affiliations:** 1 Department of Health & Physical Education, The Education University of Hong Kong, Hong Kong, China; mason-mi.zhou@connect.polyu.hk; 2 School of Science and Technology, Hong Kong Metropolitan University, Hong Kong, China; khung@hkmu.edu.hk (K.H.); s1291192@live.hkmu.edu.hk (J.G.); s1389098@live.hkmu.edu.hk (J.D.); 3 Rope Therapy Association 360 Access International Company Ltd., Hong Kong, China; marco@tafc.org.hk (M.C.-C.W.); enroll@tafc.org.hk (C.Y.-C.C.); 4 Mars CRP Academy Ltd., Hong Kong, China; enroll@ropetherapy.org (T.K.-T.C.); drbennylam@hkapsac.org (B.W.-L.L.)

**Keywords:** autism spectrum disorder, attention deficit hyperactivity disorder, sensory integration therapy, temperament traits, eye-tracking, social attention, rope therapy

## Abstract

Background: Autistic children experience social communication challenges that are often linked to reduced social motivation and attention. However, there is currently no effective intervention to improve social attention in autistic children. Objective: This study compared the effects of rope therapy (RT), a novel intervention, with traditional sensory integration therapy (SIT) on social attention and temperament traits in autistic children. Methods: A two-arm randomized controlled trial was conducted in which participants were randomly assigned to RT (*n* = 14) and SIT (*n* = 12) groups. Social attention was assessed using eye-tracking parameters, and temperament trait changes were measured using the Taylor–Johnson Temperament Analysis (T-JTA) scale. Results: Both groups showed significant improvements in social attention over time (*p* < 0.05). Although the RT group demonstrated numerically greater improvements in social attention compared to the transitional SIT group, most of the between-group differences were not statistically significant. Additionally, the RT group showed significant reductions in anxiety and emotional repression temperament traits (*p* < 0.05). Conclusions: RT exhibits promise as an effective intervention for improving social attention and temperament trait patterns in autistic children. Further research is required to confirm the findings of this study and explore the long-term effects of RT.

## 1. Introduction

Autism spectrum disorder (ASD) is a neurodevelopmental disorder that predominantly manifests in social communication challenges [1]. Social motivation theory suggests that social communication difficulties experienced by autistic children are partly caused by reduced interest and motivation relating to social stimuli, including a lower reward response to facial expressions, eye contact, and general social interaction [2]. These difficulties are also referred to as social attention impairment, which can create barriers to understanding and responding to people’s emotions and to establishing and maintaining interpersonal relationships [3,4]. Social attention impairment may also affect children’s comprehension of social context and nonverbal communication, limiting their social adaptability and engagement [5].

No consensus has been reached in the literature on which therapies should be employed to improve social attention in autistic children. Peer relationship development and joint attention interventions have been unsuccessful at improving social communication impairments, particularly when these interventions were mediated by individuals other than highly-trained university clinicians [6]. Numerous barriers can prevent the success of real-world interventions, resulting in high withdrawal rates from interventions [7]. Regarding the therapies currently used for autistic children, sensory integration therapy (SIT) has been used to improve sensory processing in such children through vestibular system stimulation and potentially enhances social functioning, orientation, and attention [8]. However, notable limitations have been reported for SIT, including lengthy therapist training durations, substantial equipment needs, extensive space requirements, high financial costs, and slow progression to observable therapeutic outcomes [9]. From a clinical standpoint, the effectiveness of SIT is diminished for children above 6 years old because of their higher weights and lower compliance, and therefore, such children require multiple therapists for SIT to be successful.

Temperament refers to biologically based individual differences in emotional reactivity, affectivity, and self-regulation that emerge early in life and remain relatively stable across development [10,11]. A substantial body of research has established temperament as a central organizer of psychological development, influencing social behavior, emotional wellbeing, and later life outcomes [12]. The latest temperament traits have been shown to correlate with sensory features and attentional functioning in autism children [13,14,15]. This association reflects shared underlying mechanisms, whereby both temperament traits and sensory-attentional profiles are supported by overlapping neural systems involved in executive attention and self-regulation. Therefore, improving sensory modulation and attentional control through structured vestibular and proprioceptive input may also help enhance temperament-related outcomes. However, this hypothesis requires further empirical validation.

Rope therapy (RT) is a body-based modality emerging from clinical and somatic psychology principles that builds upon SIT. Compared with traditional SIT, RT’s more flexible and dynamic delivery of sensory input underlies its enhanced effects [16,17]. While SIT typically provides vestibular stimulation along fixed axes (e.g., forward–backward swings or simple linear slides), RT’s dual-rope/pulley arrangement allows real-time modulation of movement direction, amplitude, and resistance [18]. This multi-vector vestibular input could engage cerebellar–vestibular and somatosensory circuits more variably than SIT, potentially accelerating adaptive changes in posture control, spatial orientation, and sustained attention [18,19]. Similarly, by grading rope tension and requiring active engagement, RT may more effectively drive proprioceptive feedback loops, leading to stronger body-schema refinement relative to the predominantly passive inputs of SIT [20,21]. RT’s combination of rhythmic loading and task complexity may further contribute to superior outcomes. The cyclical pull–release patterns inherent in rope activities might better recruit ventral vagal pathways, helping to downregulate sympathetic arousal and establish a calmer physiological state for social engagement—an effect less directly targeted by standard SIT swings.

While the theoretical foundation of RT has not yet been fully established, its hypothesized mechanisms involve sensorimotor, autonomic, and emotional integration, supported by the following frameworks:

1. Vestibular and Proprioceptive Regulation

RT activates the vestibular system through spin-based or pendular movements while stimulating proprioception via rope resistance and tension-based tasks. This dual activation is known to support postural tone, motor control, and arousal modulation [16,22].

2. Polyvagal Engagement

The guided, rope-based movements in RT mimic predictable, rhythmic patterns that signal safety to the autonomic nervous system. This promotes a shift from sympathetic arousal (fight/flight) to ventral vagal states that support social attention and emotional connection [23]. This rhythm-based approach aligns with recent research advocating somatic co-regulation over purely cognitive methods in autism interventions [23].

3. Cognitive–Motor Integration

RT exercises often require sequenced, bilateral, or reciprocal movements, activating executive function networks responsible for planning, shifting, and inhibition. By embedding physical tasks within a predictable structure, RT enables embodied cognitive rehearsal—particularly beneficial for children with ADHD comorbidity or dyspraxia.

4. Symbolic and Emotional Processing

In advanced stages, rope tasks serve as metaphors to externalize relational boundaries, inner tension, or resilience, thereby fostering emotional literacy and self–other awareness. This approach aligns with psychodynamic and narrative therapy traditions, allowing therapists to connect movement-based responses with verbal or reflective processing over time.

Since its initial development in 2014, RT has been continuously refined for clinical application and has already been implemented in regions including Hong Kong and mainland China [24]. However, there was only one study quantifying the therapeutic efficacy of RT [25]. The current pilot study was designed to explore the effect of RT on social attention and temperament in autistic children. We hypothesized that RT would be more effective than SIT in enhancing social attention and improving temperament in autistic children.

## 2. Methodology

### 2.1. Study Design

**Figure 1:**
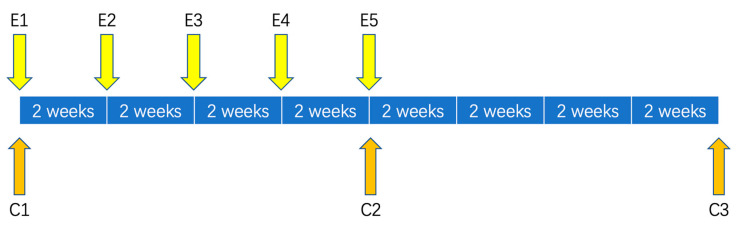
Assessment schedule. E: Eye-tracking assessment; C: T-JTA assessment.

The present quantitative study employed a two-arm randomized controlled trial design to compare the effects of RT and traditional SIT on social attention and temperament traits in autistic children. Social attention changes were considered the primary outcome measure. Commonly used eye-tracking parameters, including first fixed-duration (FFD) and total dwell time (TDT), were adopted to assess children’s social attention through eye-tracking patterns. Participants were randomly allocated into either the experimental group (RT) or control group (SIT). Eye-tracking data were collected and tested every 2 weeks for a total of five assessments (Figure 1, E1–E5). Participant caregivers completed the Taylor–Johnson Temperament Analysis (T-JTA) scale at the beginning and culmination of and at 8 weeks after the experiment (Figure 1, C1–C3).

### 2.2. Participants

Participants were recruited by employing a multichannel approach in virtual and physical settings through informational posters, social media outreach, in-person briefings, and referral systems. The screening process was conducted by two independent researchers, who provided a comprehensive outline of the study parameters for potential participants or their guardians and obtained informed consent. Regarding the inclusion criteria, participants were required to be between 6 and 18 years old and possess a clinical diagnosis of ASD either with or without a concurrent diagnosis of attention-deficit/hyperactivity disorder (ADHD), as confirmed by a qualified medical practitioner (A registered pediatrician with expertise in developmental disorders or a clinical psychologist licensed under the Hong Kong Psychological Society). Participants were deemed ineligible if they were undergoing pharmacological treatment for autism or ADHD, had been diagnosed with an ocular condition (e.g., strabismus or amblyopia) that could impair balance, or were autistic individuals with co-occurring neurological conditions other than ADHD. All participants had prior psychological evaluations confirming age-appropriate or near age-appropriate cognitive functioning. We excluded individuals with known or suspected intellectual disability (IQ < 70) based on previous educational and medical records. The study obtained ethical clearance from the Education University of Hong Kong Human Research Ethics Committee under reference no. 2022-2023-0295. Written consent was provided by participants’ parents or legal guardians prior to the experiment.

### 2.3. Intervention Design

**Figure 2:**
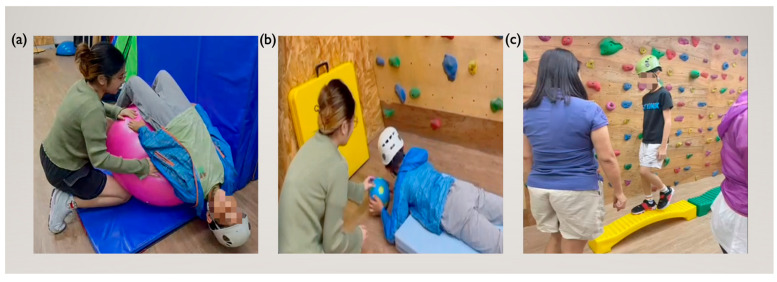
Training activities: (**a**) Bridges with an exercise ball; (**b**) prone ball push-ups; (**c**) balance beam ambulation.

The participants were required to attend biweekly training sessions for a period of 8 weeks. Each session was divided into four phases. The initial phase involved a 5-min calisthenic warm-up directed by rope therapists, which had the dual objectives of acclimating participants to the therapeutic environment and personnel and minimizing potential musculoskeletal injuries. Next, therapists supervised participants as they completed three targeted intervention activities, each lasting 3–5 min. These included bridges, prone elevations using an exercise ball, and walking along a balance beam (Figure 2). The complexity and intensity of these activities were systematically increased as the experiment progressed.

**Figure 3:**
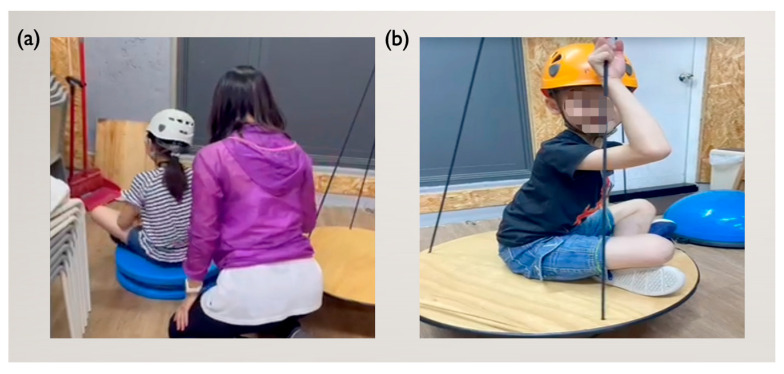
Spinning activities: (**a**) Spinning with SIT equipment; (**b**) spinning with RT equipment.

After completing these activities, participants in the control group were provided with traditional SIT equipment (Figure 3a), whereas those in the experimental group were given RT equipment (Figure 3b). Rope therapists ensured stability and proper helmet use, adjusting spinning speed and duration for clockwise and counterclockwise rotations on the basis of participant sensitivity.

Two volunteers assisted the participants in removing their helmets after training. Participants experiencing persistent dizziness received massages behind the ears or were instructed to perform multiple jumps to alleviate discomfort.

### 2.4. Outcome Measures

Data collection took place in a designated testing room containing only an adjustable-height chair and a central screen with a resolution of 1024 × 768 pixels, horizontal visual angle of 14.3 pixels/degree, and vertical visual angle of 19.1 pixels/degree. An eye-tracking system (PupilLab Neon Glass, Berlin, Germany) with an accuracy of 1.6 degrees and a sampling rate set at 200 Hz was used to monitor the participants’ eye movements. The system included a video-based head-mounted device that applied deep learning algorithms to estimate gaze position.

The participants were instructed to sit on the adjustable-height chair in front of the screen. A researcher carefully adjusted the seating and screen orientation to the participants’ eye level, ensuring their heads were directly facing the center of the screen. Another researcher assisted the participants in putting on Neon glasses, which were calibrated using a five-point manual calibration. Participants were asked to look sequentially at the centers of five white crosses displayed at the four corners and center of the screen for 5 s both before and after the assessment. Those who did not properly adhere to the calibration procedure were excluded from the experiment.

### 2.5. Stimulus Design

A video stimulus was developed on the basis of the preferential looking paradigm, which is commonly used to assess perceptual abilities and social attention in nonverbal participants, such as infants [26,27].

**Figure 4:**
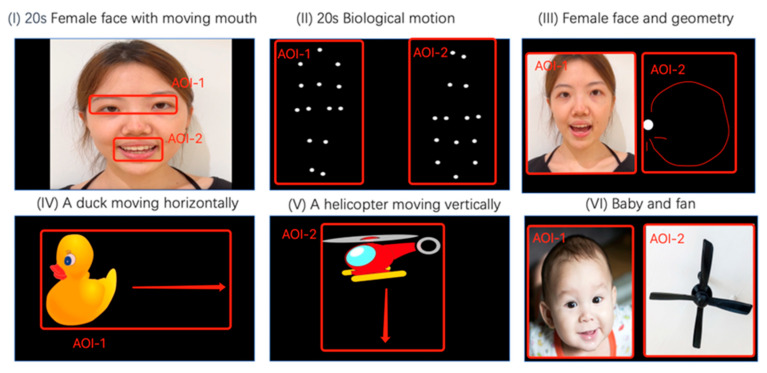
Video stimuli part 1. AOI: Area of Interest.

Stimulation was divided into two stages. During the first stage (Figure 4), eye-tracking was assessed for 90 s on the basis of six paradigms, with each including two areas of interest (AOIs): social and nonsocial [28]. Social and nonsocial stimulus AOIs are marked with the suffixes 1 and 2, respectively.

1. Paradigm I (20 s): A woman’s face silently mouthing the alphabet was displayed. Attention to the eyes (AOI-1) and mouth (AOI-2) reflects social communication and language development [29].
2. Paradigm II (20 s): A walking figure, both upright (AOI-1) and inverted (AOI-2), was presented in point-light display to assess attention to biological motion, which is typically diminished in autistic children, reflecting social communication and language development [29].
3. Paradigm III (20 s): The same face from Paradigm I (AOI-1) was displayed alongside a moving dot (AOI-2) to test preference for social versus moving stimuli [29].
4. Paradigm IV and V (20 s): A moving duck (AOI-1) and a helicopter (AOI-2) were displayed to assess sustained attention to animals versus objects [28].
5. Paradigm VI (10 s): A baby’s face (AOI-1) was displayed next to an electronic fan (AOI-2) to evaluate preference for faces versus objects [28].

**Figure 5:**
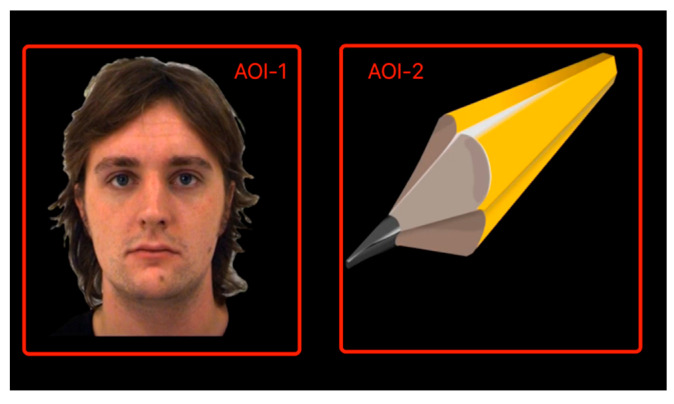
Video Stimuli Part 2. AOI: Area of Interest.

The second stage (Figure 5) lasted 90 s, and in it, eye-tracking paradigms were used that paired social and nonsocial images to assess visual attention to faces and objects by following the method of Sasson and Touchstone [30].

Eighteen social images of faces, balanced for gender, were taken from the Amsterdam Collection of Dynamic Facial Expressions [31]. Two types of nonsocial images were collected from Pixabay: circumscribed interest objects (CIO), such as toys, vehicles, and animals, and noncircumscribed interest objects (NCIO), such as plants, furniture, and tools. A preference for nonsocial objects over faces is typically considered a marker of ASD [3,32,33].

### 2.6. Taylor–Johnson Temperament Analysis

The T-JTA scale is a widely used tool for assessing nine personality traits, providing insights into an individual’s temperament and behavior [34]. While there are no known peer-reviewed studies that directly link the T-JTA to the prediction of anxiety, social withdrawal, or academic performance in children or adolescents, the instrument has long been recognized for its broad application in assessing personality traits relevant to emotional stability, sociability, and self-esteem across adolescent and adult populations [35]. These traits—particularly emotional stability and introversion–extraversion—have been well documented in developmental psychology as correlating with adolescent outcomes such as anxiety, peer relationships, and academic engagement.

Given that the T-JTA measures temperament domains comparable to those assessed in other validated tools, it holds potential utility for identifying adolescents who may be at risk for social and emotional difficulties. In the present study, the T-JTA was used to track changes across multiple assessments in nine personality traits: nervous, depressive, active-social, expressive-responsive, sympathetic, subjective, dominant, hostile, and self-disciplined. These traits can form temperament patterns such as dominance/hostility, anxiety, emotional repression, and withdrawal [36,37]. The T-JTA also includes subscales for personality traits such as emotional stability, self-esteem, and interpersonal effectiveness. Assessment was further refined through supplemental scales with Sten scores ranging from extremely low (1) to extremely high (10).

### 2.7. Data Processing

A customized program was used to screen the experimental data. If a participant was fixated on the screen for less than 50% of a test, the entire test was deemed low-quality and discarded, in alignment with previous studies [27,38,39,40]. All data for participants with two or more low-quality tests were excluded from statistical analysis. Due to the inability of the Pupil Cloud eye-tracking software (Version v6.0) in the Neon device to select AOIs in dynamic videos, particularly when participants exhibited considerable head movement, AOIs and fixations were determined manually for these videos to ensure accuracy. Two trained psychology researchers with relevant expertise counted the fixation points within each paradigm and compiled them into tables. Any discrepancies between their results were resolved through review and confirmation by a third senior researcher.

### 2.8. Statistical Analysis

Statistical analysis was conducted using SPSS version 28.0 (IBM Corp., Armonk, New York, NJ, USA) with the significance level set at 0.05. To reduce the effect of confounding variables on the results, the demographic characteristics of the two groups were compared using independent sample *t*-tests and Chi-square tests.

A two-way repeated measure analysis of variance (ANOVA) was used to examine the effects of group × time interaction on the aforementioned parameters. Results were reported using mean (m) and standard deviation values, *p* values (*p*), and equality of variances (F) values. Post hoc tests were used to identify any differences from *p* values. Before data analysis, Kolmogorov–Smirnov and Levene’s tests were performed to assess the normality and homogeneity of variances, respectively, and Mauchly’s test was performed to assess the assumption of sphericity. If the assumption was not met, the Greenhouse–Geisser correction was used to make adjustments. Results for eye-gazing parameters were plotted according to the trend of both groups with a linear regression model using R (Version 4.4.3, R Foundation for Statistical Computing, Vienna, Austria) [41].

## 3. Results

### 3.1. Demographic Characteristics

A total of 29 participants were enrolled in the study. Two participants from the SIT group voluntarily withdrew, and one additional participant was eliminated because of low-quality data. This left a final sample size of 26. The overall data loss rate was 10.3%. The demographic characteristics of the participants are presented in Table 1. No significant age or gender distribution differences were observed between the two groups.

### 3.2. Temperament Traits

The baseline results of the temperament trait assessment for both groups are displayed in Table 2. Two participants in the RT group did not take the T-JTA test; therefore, temperament trait patterns were collected from only 12 participants in this group. The RT group exhibited greater temperament trait diversity than the SIT group did.

### 3.3. Social Attention

**Figure 6:**
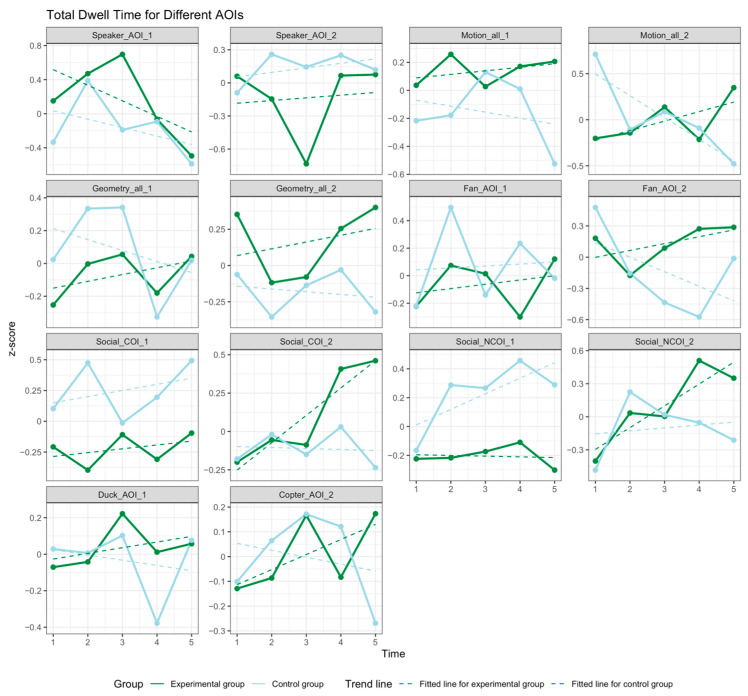
Changes in TDT for different AOIs.

Figure 6 illustrates the TDT results. The TDT for social stimuli increased as treatment progressed for both groups, with it particularly exhibiting an increase before the third assessment, after which the TDT fluctuated. Although the differences for most AOIs both within and between groups were nonsignificant, the RT group generally demonstrated a higher and more stable TDT for social stimulus AOIs over time. The TDT for nonsocial stimulus AOIs in the RT group also increased with time. By contrast, in the SIT group, the TDT for nonsocial stimulus AOIs exhibited a generally flat or declining trend.

As illustrated in Table A1 of Appendix A, within-group comparison revealed TDT improvements across nearly all AOIs in both groups following treatment. However, with the exception of AOI 1 in Paradigm VI (Fan AOI 1), no significant time effects were observed. The between-group comparison indicated that the RT group exhibited greater changes in most AOIs compared with the SIT group, although the majority of the differences between groups were not significant. Significant differences were noted in only a few AOIs: Social_NCOI_1 (F (1111) = 5.674, *p* = 0.019), Social COI 1 (F (1111) = 7.091, *p* = 0.009), Geometry_AOI_2 (F (1111) = 3.564, *p* = 0.062), and Speaker_AOI_1 (F (1111) = 3.259, *p* = 0.074).

Post hoc tests were performed to further examine these differences. For Social_NCOI_1, the pairwise comparison did not reveal significant differences between the two groups. Nevertheless, these differences gradually increased as training progressed (Time 1: t = 0.162, *p* = 0.87; Time 5: t = 1.465, *p* = 0.146). For Social_COI_1, no significant difference was observed in either group at Time 1 (t = 0.802, *p* = 0.424). However, a significant difference emerged after 2 weeks of training at Time 2 (t = 2.177, *p* = 0.032). Although the gap narrowed at Time 3, the RT group consistently exhibited lower attention to the COI object than the SIT group did during the assessments at Time 4 and Time 5. For Speaker_AOI_1, no significant difference was observed between groups at Time 1 (t = −1.277, *p* = 0.204). However, a significant difference emerged after 3 weeks of training (Time 3: t = −2.318, *p* = 0.022). For Geometry AOI 2, no significant difference was observed between groups at Time 1 (t = −1.023, *p* = 0.309), but a significant difference emerged after 2 months of training (Time 5: t = −1.757, *p* = 0.082).

**Figure 7:**
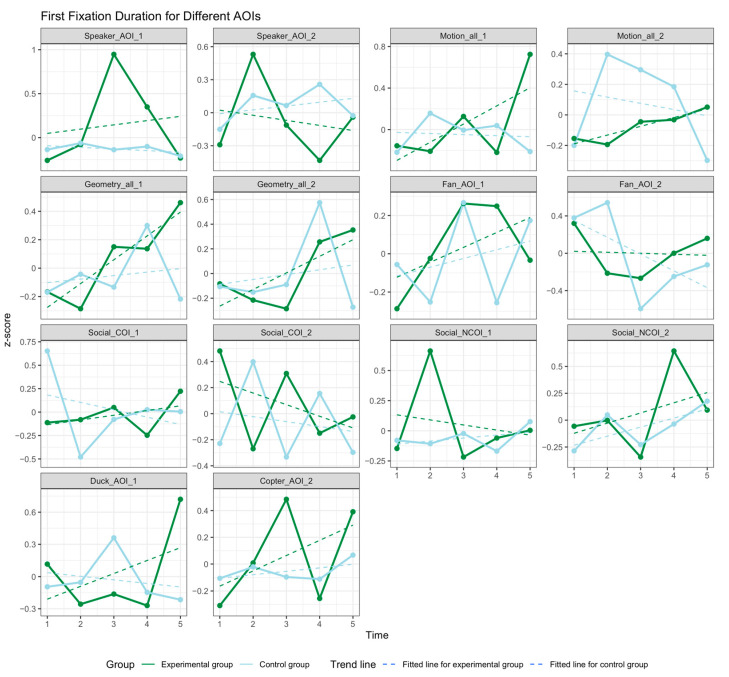
Changes in FFD for different AOIs.

Figure 7 presents the FFD results. The fixation durations for social stimulus AOIs (suffix 1) tended to increase over time in both groups. The RT group exhibited greater interest in more social stimulus AOIs than the SIT group did. For nonsocial stimulus AOIs (suffix 2), the FFD for both groups fluctuated over time. In the RT group, the FFD decreased for Speaker_AOI_2, Social_COI_2, and Fan_AOI_2 as treatment progressed and increased for the remaining paradigms. In the SIT group, the FFD decreased for Social_COI_2, Fan_AOI_2, and Motion_AOI_2 and increased in all other paradigms as treatment progressed.

As displayed in Table A2 of Appendix A, the within-group comparison revealed FFD improvements for nearly all AOIs in both groups following treatment, although most of these changes were not significant. Significant differences were observed for Copter AOI 2 (F (1105) = 3.406, *p* = 0.012). Post hoc tests were performed to further examine within-group differences. The results indicated a significant difference between Time 1 and Time 5 in the RT group (t = −2.741, *p* = 0.055), indicating a notable increase in the time spent observing the helicopter as treatment progressed. By contrast, no significant changes were observed between time points in the SIT group.

The between-group comparison indicated that the RT group exhibited greater changes for most AOIs relative to the SIT group. However, none of the differences between groups were significant.

### 3.4. Temperament Trait Pattern Changes

As displayed in Table 3, the temperament trait patterns of the participants in the SIT group exhibited no significant changes from before to after treatment, with the anxiety pattern consistently present throughout. Several participants who initially exhibited no significant temperament trait patterns developed new patterns following treatment, such as anxiety or emotional repression. Some participants continued to exhibit the emotional repression pattern between the second and third assessments. For others, complex combinations of dominant/hostile/indifferent, patterns emerged by the third assessment. These results suggest an overall deterioration in temperament traits, in which initial improvements (if any) did not persist, and more negative patterns began to dominate, indicating that SIT treatment may not have produced lasting positive effects in the control group.

As indicated in Table 4, the temperament trait patterns of the participants in the RT group exhibited significant improvement after treatment. Overall, previously identified patterns of withdrawal, emotional repression, anxiety, and similar tendencies either became less severe (e.g., participants exhibited only emotional repression or anxiety) or completely disappeared by the second assessment, indicating that treatment produced sustained positive outcomes. However, some patterns that had disappeared after treatment re-emerged during the follow-up period. For instance, one participant whose pattern of emotional repression disappeared following treatment had this pattern re-emerge by the third assessment, accompanied by additional dominant, hostile, inhibited, subjective, impulsive, and indifferent patterns.

As displayed in Table A3 of Appendix A, both groups exhibited overall improvements across T-JTA assessment scales. However, significant improvements were only observed for the interpersonal effectiveness and alienating scales.

A significant time effect was observed for the interpersonal effectiveness scale (F (2,44) = 2.777, *p* = 0.029, ηp^2^ = 0.149), indicating significant score changes over time. Post-hoc analysis revealed that interpersonal effectiveness improved significantly in the RT group following treatment. This scale measured the ability to interact successfully with others, with higher scores indicating improved communication skills, empathy, and conflict resolution abilities.

A significant time effect was also observed for the alienation scale (F (2,44) = 1.946, *p* = 0.017, ηp^2^ = 0.168). Post hoc analysis revealed that alienation scores in the RT group decreased significantly following treatment. This scale was used to assess feelings of isolation or detachment, with higher scores indicating greater difficulty in forming connections and feeling integrated within social groups.

## 4. Discussion

The present study compared the effects of traditional SIT and RT, a new intervention, on social attention in autistic children. The participants’ attention to social and nonsocial stimuli was measured using eye-tracking devices based on two common indicators: TDT and FFD. To the authors’ knowledge, this is the first study to investigate the therapeutic effects of RT on autistic children. The results suggested that both SIT and RT had significant therapeutic effects. Following the 8-week intervention, both groups showed increased attention to social stimuli, with more pronounced effects in the RT group. However, most experimental paradigms revealed no statistically significant between-group differences.

Previous studies have established the effectiveness of SIT in enhancing social functioning in autistic children [8,42,43]. The results showed significant improvements in social interaction, motor function, and tactile performance in the SIT group, with effects maintained at a two-month follow-up. These findings align with our results. However, few studies have specifically investigated SIT’s effects on social attention. Our findings suggest that SIT may significantly impact social attention, potentially through improving selective attention to sensory inputs and enhancing overall sensory processing capabilities. Such improvements could help children better filter and respond to sensory stimuli, enabling more accurate identification of socially relevant cues in complex environments. Supporting this mechanism, a neuroimaging study found that SIT enhances neural activation in brain regions involved in both sensory processing and executive function [44], providing a plausible neurological basis for SIT’s effects on social attention.

RT delivers multidirectional, multi-intensity vestibular stimulation to participants. The previous research demonstrates that the vestibular system influences cognitive function—particularly visuospatial abilities, attention, and executive function [45]—with evidence suggesting that complex or varied vestibular stimulation enhances performance in demanding information-processing tasks [46]. Consistent with this rationale, our observations revealed that RT participants showed greater improvement in locating and attending to social stimuli compared to the SIT group. Notably, RT did not enhance attention to nonsocial objects over time, potentially reflecting either a preferential allocation of attentional resources toward social information or habituation to nonsocial stimuli.

The RT group exhibited significant improvements in temperament traits post intervention, while the SIT group showed no comparable changes. This suggests that RT may be more effective than traditional SIT in addressing specific maladaptive emotional patterns, including anxiety and emotional repression. A plausible mechanism is RT’s capacity to provide a broader, more dynamic range of sensory input. By integrating diverse vestibular stimuli—engaging both linear and rotational movement systems—RT may exert stronger regulatory effects on the autonomic nervous system, thereby promoting emotional stability and reducing arousal-related distress. In contrast, traditional SIT’s more limited and repetitive vestibular stimulation may be less effective in fostering emotional resilience.

Individual differences appeared to play a crucial role in treatment outcomes. Different responses to therapy among participants may suggest that some individuals require more time than others to develop and sustain new behavioral patterns, contributing to the re-emergence of negative emotional patterns. Additionally, following treatment, participants returned to preexisting environments that lacked the necessary support to maintain the positive changes achieved during therapy. Environmental stressors or triggers may have contributed to the resurgence of negative emotional patterns. Therefore, further research is required to evaluate the long-term efficacy of RT. Extending the intervention duration and implementing longer follow-up assessments would provide a better understanding of the persistence of treatment effects. Moreover, given the individual differences observed in responses to RT, future studies should investigate the personal factors (such as age, symptom severity, and family support) that influence treatment outcomes and develop personalized interventions accordingly.

To the authors’ knowledge, the present study is the first to use the PupilLab Neon device in ASD research. Other eye-tracking devices require participants to follow instructions and complete pre-experiment calibration to ensure data accuracy. However, noncompliance among autistic children can cause challenges when eye-tracking experiments are conducted. The “wear-and-use” and calibration-free features of the Neon device are suitable for eye-tracking research in autistic children. In the present study, the time required for device adjustment was brief, averaging less than 5 min from the child’s arrival at the test site to the start of the experiment, which enabled therapy to be completed quickly. The device also has automatic adjustment capabilities. When a participant’s head movement caused the eye-tracking device to deviate from its original position, the built-in gyroscope automatically adjusted for potential errors, improving the accuracy of the experimental data. These device features can enable large-sample eye-tracking experiments among children with special education needs. However, because of flaws in the software of the Neon device, data collection and processing required considerable effort. The methods section provides a detailed report on the data collection and processing steps, which can serve as a reference for future researchers using the Neon device.

### 4.1. Study Limitations and Future Research

The present study had several limitations. First, the relatively short intervention duration of 8 weeks prevented assessment of long-term RT efficacy, which can be evaluated by future studies with longer durations. Second, although the Neon eye-tracking device is sufficiently adaptable for experiments with children, in the current study, participants’ heads were not immobilized during the experiment, resulting in data inaccuracies due to head movements. Future researchers using the Neon device in experiments with children should consider immobilizing participants’ heads by, for example, fixing the Neon glasses on a stable frame. This approach could resolve data inaccuracies caused by head movement and the time-consuming calibration process. Third, T-JTA has not been validated in autistic children; future psychometric research is needed to validate temperament measures in this population.

Additionally, the study included only 26 participants in the final data analysis—a sample size too small to draw definitive conclusions; larger-scale trials are therefore needed to confirm RT’s effectiveness. Moreover, only three of those participants were female. Although we controlled for gender when evaluating RT efficacy, the very low number of girls limits the generalizability of our findings and underscores the need for further research specifically examining RT’s impact on autistic female children.

Finally, it is important to note that this investigation was a pilot study designed to explore RT’s therapeutic potential. The theoretical framework underpinning RT remains underdeveloped: while we have outlined several working hypotheses, each must be rigorously tested and validated in subsequent, more comprehensive studies.

### 4.2. Implications

The present pilot findings suggest that RT may serve as a feasible alternative to traditional SIT for autistic children, offering a more adaptable sensorimotor framework without requiring extensive equipment or space. Its relative portability and potential for home-based delivery may increase accessibility and continuity of care. However, before recommending RT as a routine replacement for SIT, larger trials with longer intervention periods and follow-up are needed to confirm these preliminary observations.

## 5. Conclusions

The findings of the present study provide preliminary evidence supporting the effectiveness of RT in improving social attention and temperament traits in autistic children. Although RT demonstrated numerically greater improvements in social attention, engagement, and reductions in negative emotional patterns compared to traditional SIT, these differences were not statistically significant. The durability of these effects remains uncertain, and additional studies are required that incorporate extended intervention durations and follow-up assessments. Future research should focus on larger, more diverse samples to validate these findings.

## Figures and Tables

**Table 1:** Demographic characteristics of both groups.

Stratification	Levels	SIT Group (*n* = 12)	RT Group (*n* = 14)	*p*
Age	Mean ± SD	7.3 ± 1.4	8.9 ± 3.5	0.134
Gender	Female	2 (16.7%)	1 (7.1%)	0.887
	Male	10 (83.3%)	13 (92.9%)	
Comorbidity	ADHD	2 (16.7%)	4 (28.6%)	0.733
	Developmental Delay	3 (25%)	4 (28.6%)	
	Intellectual Disability	1 (8.3%)	1 (7.1%)	
	ADHD/Developmental Delay	1 (8.3%)	0 (0%)	
	ADHD/Intellectual Disability	1 (8.3%)	0 (0%)	
	No Comorbidity	4 (33.3%)	5 (35.7%)	

**Table 2:** Temperament trait patterns of both groups.

Temperament Trait Pattern	SIT Group (*n* = 12)	RT Group (*n* = 12)
None	6	5
Anxiety, Dependent/Hostile	2	0
Anxiety, Emotionally Repressed	2	2
Anxiety, Dominant/Hostile/Subjective/Impulsive/Indifferent	1	1
Anxiety, Emotionally Repressed, Dependent/Hostile	1	0
Withdraw, Emotionally Repressed	0	1
Anxiety	0	1
Dominant/Hostile/Subjective/Impulsive/Indifferent	0	1
Emotionally Repressed	0	1

**Table 3:** Temperament trait pattern changes in SIT group.

1st Assessment	2nd Assessment	3rd Assessment
None	Emotionally Repressed	None
None	Emotionally Repressed	Emotionally Repressed
None	None	None
None	Anxiety, Emotionally Repressed	Anxiety
None	Withdrawal, Emotionally Repressed	None
Anxiety, Dominant/Hostile/Subjective/Impulsive/Indifferent	Anxiety, Dominant/Hostile/Subjective/Impulsive/Indifferent	None
Anxiety, Dependent/Hostile	Anxiety	Dominant/Hostile/Indifferent
Anxiety, Dependent/Hostile	None	None
Anxiety, Emotionally Repressed, Dependent/Hostile	Anxiety, Emotionally Repressed	Anxiety, Emotionally Repressed
Anxiety, Emotionally Repressed	Anxiety, Emotionally Repressed	Anxiety
Anxiety, Emotionally Repressed	None	None
None	None	None

**Table 4:** Temperament trait pattern changes in RT group.

1st Assessment	2nd Assessment	3rd Assessment
Withdraw, Emotionally Repressed	Emotionally Repressed	None
Anxiety	None	None
None	None	None
None	None	None
None	None	None
Dominant/Hostile/Subjective/Impulsive/Indifferent	None	Dominant/Hostile/Impulsive/Indifferent
Anxiety, Dominant/Hostile/Subjective/Impulsive/Indifferent	Anxiety, Dominant/Hostile/Subjective/Impulsive/Indifferent	Anxiety, Dominant/Hostile/Subjective/Impulsive/Indifferent
None	None	None
None	Dominant/Hostile/Subjective/Impulsive/Indifferent	None
Anxiety, Emotionally Repressed	None	Anxiety
Anxiety, Emotionally Repressed	Anxiety	Anxiety, Emotionally Repressed, Dominant/Hostile/Inhibited/Subjective/Impulsive/Indifferent
Emotionally Repressed	Withdrawal, Emotionally Repressed	Emotionally Repressed

## Data Availability

The original contributions presented in this study are included in the article/Appendix A. Further inquiries can be directed to the corresponding author.

## Appendix A

**Table A1:** Total dwell time two-way repeated measure ANOVA results.

AOI	Effect	Df	Mean Square	F	*p*
Speaker_1	Group	1	30,575,193	3.259	0.074 *
	Time	4	5,554,721	0.592	0.669
	Group: Time	4	9,065,489	0.966	0.429
	Error	111	9,380,645		
Speaker_2	Group	1	47,061,800	2.265	0.135
	Time	4	9,879,947	0.476	0.754
	Group: Time	4	19,595,296	0.943	0.442
	Error	111	20,777,333		
Duck_1	Group	1	1,239,590	0.137	0.712
	Time	4	5,806,979	0.640	0.635
	Group: Time	4	1,962,447	0.216	0.929
	Error	111	9,074,688		
Copter_2	Group	1	41,990	0.004	0.950
	Time	4	2,560,020	0.237	0.917
	Group: Time	4	4,433,926	0.411	0.800
	Error	111	10,787,828		
Fan_1	Group	1	3,592,540	0.597	0.441
	Time	4	14,145,420	2.351	0.058 *
	Group: Time	4	3,743,333	0.622	0.648
	Error	111	6,016,053		
Fan_2	Group	1	10,799,313	2.159	0.145
	Time	4	5,104,847	1.021	0.400
	Group: Time	4	6,332,881	1.266	0.288
	Error	111	5,001,615		
Motion_1	Group	1	53,437,934	2.708	0.103
	Time	4	9,610,745	0.487	0.745
	Group: Time	4	10,482,561	0.531	0.713
	Error	111	19,736,405		
Motion_2	Group	1	827,789	0.069	0.793
	Time	4	13,660,345	1.139	0.342
	Group: Time	4	30,112,539	2.511	0.046 **
	Error	111	11,992,811		
Geometry_1	Group	1	12,480,465	0.635	0.427
	Time	4	3,711,877	0.189	0.944
	Group: Time	4	5,572,368	0.284	0.888
	Error	111	19,652,160		
Geometry_2	Group	1	68,993,915	3.564	0.062 *
	Time	4	2,062,186	0.107	0.980
	Group: Time	4	6,714,346	0.347	0.846
	Error	111	19,356,268		
Social_COI_1	Group	1	411,490,489	7.091	0.009 ***
	Time	4	49,535,009	0.854	0.494
	Group: Time	4	30,530,272	0.526	0.717
	Error	111	58,027,302		
Social_COI_2	Group	1	96,141,444	1.480	0.226
	Time	4	43,640,569	0.672	0.613
	Group: Time	4	42,639,732	0.656	0.624
	Error	111	64,970,748		
Social_NCOI_1	Group	1	345,392,987	5.674	0.019 **
	Time	4	53,553,197	0.880	0.479
	Group: Time	4	17,251,416	0.283	0.888
	Error	111	60,876,216		
Social_NCOI_2	Group	1	79,075,380	1.324	0.252
	Time	4	22,361,182	0.374	0.827
	Group: Time	4	46,164,060	0.773	0.545
	Error	111	59,746,994		

Notes: * Represents a significant difference (*p* < 0.1); ** Represents a significant difference (*p* < 0.05); *** Represents a significant difference (*p* < 0.01).

**Table A2:** First fixation duration two-way repeated measure ANOVA results.

AOI	Effect	Df	Mean Square	F Value	*p* Value
Speaker_1	Group	1	3,347,642	2.09	0.151
	Time	4	2,377,236	1.484	0.212
	Group: Time	4	2,016,773	1.259	0.291
	Residuals	105	1,601,620		
Speaker_2	Group	1	374,223	0.434	0.511
	Time	4	1,237,435	1.437	0.227
	Group: Time	4	750,427	0.871	0.484
	Residuals	105	861,291		
Duck_1	Group	1	119,833	0.109	0.742
	Time	4	1,395,261	1.268	0.287
	Group: Time	4	1,716,430	1.56	0.191
	Residuals	105	1,100,450		
Copter_2	Group	1	220,376	0.505	0.4788
	Time	4	1,485,896	3.406	0.0116 **
	Group: Time	4	372,462	0.854	0.4943
	Residuals	105	436,198		
Fan_1	Group	1	69,065	0.122	0.728
	Time	4	187,239	0.33	0.857
	Group: Time	4	321,836	0.567	0.687
	Residuals	105	567,149		
Fan_2	Group	1	1090	0.011	0.918
	Time	4	120,350	1.165	0.331
	Group: Time	4	138,500	1.34	0.26
	Residuals	105	103,347		
Motion_1	Group	1	1,147,740	0.27	0.604
	Time	4	1,853,803	0.436	0.782
	Group: Time	4	6,048,872	1.423	0.231
	Residuals	105	4,250,001		
Motion_2	Group	1	1,245,692	0.577	0.449
	Time	4	483,540	0.224	0.924
	Group: Time	4	1,419,753	0.658	0.623
	Residuals	105	2,157,473		
Geometry_1	Group	1	553,315	0.27	0.604
	Time	4	2,208,905	1.078	0.371
	Group: Time	4	1,379,447	0.673	0.612
	Residuals	105	2,049,865		
Geometry_2	Group	1	17,489	0.007	0.932
	Time	4	207,089	0.086	0.987
	Group: Time	4	1,979,743	0.823	0.514
	Residuals	105	2,406,820		
Social_COI_1	Group	1	16,057	0.133	0.716
	Time	4	65,201	0.541	0.706
	Group: Time	4	154,331	1.281	0.282
	Residuals	105	120,511		
Social_COI_2	Group	1	25,614	0.384	0.5369
	Time	4	26,254	0.393	0.8129
	Group: Time	4	150,268	2.252	0.0684 *
	Residuals	105	66,724		
Social_NCOI_1	Group	1	1,012,909	0.4	0.529
	Time	4	3,356,164	1.324	0.266
	Group: Time	4	2,153,334	0.85	0.497
	Residuals	105	2,534,193		
Social_NCOI_2	Group	1	75,918	0.558	0.457
	Time	4	84,495	0.622	0.648
	Group: Time	4	85,799	0.631	0.641
	Residuals	105	135,947		

Notes: * Represents a significant difference (*p* < 0.1); ** Represents a significant difference (*p* < 0.05);

**Table A3:** Two-way repeated measure ANOVA results for T-JTA subscale scores.

Scale	Source	Df	F	*p*-Value	Partial Eta Squared
**1. Overall Adjustment**	Group	1, 22	0.006	0.964	0.000
	Time	2, 44	2.528	0.091	0.103
	Time × Group	2, 44	2.196	0.126	0.091
**2. Self-Esteem**	Group	1, 22	0.248	0.623	0.011
	Time	2, 44	0.935	0.384	0.043
	Time × Group	2, 44	0.935	0.384	0.043
**3. Outgoing/Gregarious**	Group	1, 22	0.401	0.533	0.018
	Time	2, 44	0.573	0.629	0.028
	Time × Group	2, 44	0.240	0.770	0.012
**4. Interpersonal Effectiveness**	Group	1, 22	0.259	0.616	0.012
	Time	2, 44	2.777	0.029	0.149
	Time × Group	2, 44	1.527	0.233	0.088
**5. Alienating**	Group	1, 22	0.397	0.535	0.018
	Time	2, 44	1.946	0.017	0.168
	Time × Group	2, 44	1.072	0.351	0.094
**6. Industrious/Persevering**	Group	1, 22	0.001	0.974	0.000
	Time	2, 44	1.765	0.166	0.078
	Time × Group	2, 44	0.765	0.451	0.036
**7. Persuasive/Influential**	Group	1, 22	0.187	0.669	0.008
	Time	2, 44	0.146	0.864	0.007
	Time × Group	2, 44	0.229	0.796	0.010
